# Correlation Between the Magnetic Properties of Ce-Containing Magnets and the CeFe_2_ Phase at Various Sintering Temperatures

**DOI:** 10.3390/ma17225517

**Published:** 2024-11-12

**Authors:** Qingpeng Shen, Munan Yang, Ihor Bulyk, Sangen Luo, Honglong Yang, Yifan Wang, Xiaoqiang Yu, Shuwei Zhong

**Affiliations:** 1 School of Materials Science and Engineering, Jiangxi University of Science and Technology, Ganzhou 341000, China; shenqpshell@163.com (Q.S.); i.bulyk@jxust.edu.cn (I.B.); gtwy-yhl@139.com (H.Y.); 2 Key Laboratory of Development and Application of Ionic Rare Earth Resources, Ministry of Education, Ganzhou 341000, China; 3 National Rare Earth Functional Material Innovation Center & Guorui Scientific Innovation Rare Earth Functional Materials Co., Ltd., Ganzhou 341000, China; 4 College of Rare Earths, Jiangxi University of Science and Technology, Ganzhou 341000, China; sangenluo@outlook.com (S.L.); wyf18770835880@163.com (Y.W.); zhongshuweidc@163.com (S.Z.)

**Keywords:** CeFe_2_ phase, Ce_2_Fe_17_ phase, liquid phase sintering, magnetic property

## Abstract

This article investigates the relationship between the magnetic properties of magnets and the percentage and distribution of the CeFe_2_ phase at different sintering temperatures. At the lower sintering temperature, the grain boundary phase flow of the magnet is poor, more hole defects are generated in the magnet, and the comprehensive magnetic properties of the magnet are poor. An increase in sintering temperature increases the ratio of CeFe_2_ phase, improves the fluidity of grain boundary liquid phase, fills the hole defects and causes an increase in remanence. However, an increase in grain size also inhibits the coercivity of the magnet at this temperature. When the sintering temperature reaches 1080 °C, the CeFe_2_ phase ratio continues to increase, providing more liquid phase. The phase Ce_2_Fe_17_ was also decomposed into liquid phase, the continuity and wettability of grain boundary phase were optimized, and the coercivity reached a maximum of 13.18 kOe. However, the orientation of the magnet changed and the proportion of the main phase decreased, resulting in a slight decrease in the remanence (*B*_r_ = 13.17 kGs).

## 1. Introduction

Magnetic materials, as one of the important functional materials, have an influential position in modern society [1]. In recent years, the replacement of Pr and Nd in sintered Nd-Fe-B permanent magnets with highly abundant rare-earth elements (REEs) has received widespread attention due to the soaring prices of REEs Pr and Nd and the large backlog of highly abundant REEs [2,3,4,5,6,7]. With regard to the highly abundant rare-earth elements, the stability of the La_2_Fe_14_B phase is not as good as that of Ce_2_Fe_14_B and Y_2_Fe_14_B due to the larger atomic radius of La and its higher activity, which is easy to be combined with oxygen, and the price of Y is much higher than that of Ce, so it is more cost-effective to choose Ce to partially replace Pr and Nd [2,8,9,10,11,12]. However, it is worth noting that the endowed magnetic properties, i.e., magnetic polarization strength and magnetocrystalline anisotropy field, of Ce_2_Fe_14_B are significantly lower than those of Nd_2_Fe_14_B [13,14,15,16]. Therefore, the enhancement of magnetic properties of Ce-containing magnets through composition and process optimization has become a hot spot for current research.

To date, a great deal of research has been conducted on the preparation of Ce-containing sintered permanent magnets. Li et al. [17] found that Ce_2_Fe_14_B has a low melting point, and selecting an appropriate sintering temperature can optimize the magnet density and grain boundary wettability, thus improving the magnetic properties of magnets. Chen et al. [18] showed that when the Ce substitution amount in the magnet is 5–10%, the precipitated CeFe_2_ phase at the grain boundary makes the grain boundary clearer and thicker, which is conducive to the magnetic decoupling of the main phase grains and increases the coercivity of the magnet. In addition to this, some researchers have also studied the modulation of the ratio and distribution of the REFe_2_ phase in Ce-containing magnets to optimize the magnet performance [3,19,20,21,22]. Zhang et al. [23] found that the melting of the REFe_2_ phase in the liquid-phase sintering process caused an increase in liquid-phase volume fraction, thus improving the phase continuity at the grain boundary and increasing the coercivity of magnets, indicating the direction for strengthening the coercivity of sintered Ce-containing magnets. However, there is a lack of studies on the correlation between the REFe_2_ phase and the sintering temperature.

Based on Ce-containing sintered NdFeB magnets, this study systematically analyzed the connection between liquid-phase sintering temperature and the REFe_2_ phase by regulating the liquid-phase sintering temperature, which in turn affects the liquid-phase generation and flow, and combines with the magnetic properties and microstructure of the organization so as to enrich and improve the basic research theory on the industrialization of sintered Ce magnets.

## 2. Materials and Methods

### 2.1. Experimental Procedure

The raw materials were Pr-Nd alloy (Pr_25_Nd_75_, wt.%), Fe-B alloy (Fe_80_B_20_, wt.%), commercial purity iron, Ce, Co, Cu, Al, Ga, Zr, and Ti. Among them, the purity of both alloys and metals was greater than 99.9%, and all were purchased from Ganzhou Yingke Xinchuang Technology Co. Ltd. (Ganzhou, China). First, the sintered magnets with a nominal composition of Pr_5.8325_Nd_17.4975_Ce_6.67_Co_0.8_Cu_0.3_ Al_0.5_Ga_0.2_Zr_0.15_Ti_0.1_Fe_67.04_B_0.91_ (wt.%) were prepared using the strip cast (SC) technique. Then, the SC alloy was subjected to hydrogen decrepitation (HD) and jet milling (JM) to obtain powders with an average particle size of 2.8 μm. The powders were compacted at a magnetic field of 2 T and cold-isostatic-pressed at a pressure of 200 MPa. The compacted greens were sintered at 1030 to 1080 °C for 4 h, and then consecutively annealed at 890 °C for 3 h and 465 °C for 4 h in vacuum.

### 2.2. Analytical Techniques

Cylinder samples with a diameter of 10 mm and height of 6 mm were made using a wire electrical discharge cutting machine. The density of the magnet was determined using Archimedes’ method. The magnetic properties of each magnet were measured at 20 °C using a magnetic measurement system (NIM-500C, National institute of metrology, China, Beijing) designed by the China Academy of Metrology and Science. The microstructure morphology of the magnets was observed using scanning electron microscopy (SEM MIRA3-LMH, TSECAN, China, Shanghai). Differential scanning calorimetry (DSC) curves were obtained using a differential thermal analyzer (DTA, Netzsch 449F3 Jupiter, NETZSCH, China, Shanghai) in an argon environment to determine the heat absorption and excretion of each phase of the magnet at a temperature increase rate of 5 °C/min and a temperature decrease rate of 20 °C/min. X-ray diffraction (XRD-Panalytical Empyrean, Malvern Panalytical, China, Shanghai) was used to analyze the phase composition of the magnet bulk and powder samples. The samples were analyzed for elements using an electron probe microanalyzer (EPMA-JXA-8100, Japan Electronics Co., Ltd., China, Beijing).

## 3. Results and Discussion

**Figure 1:**
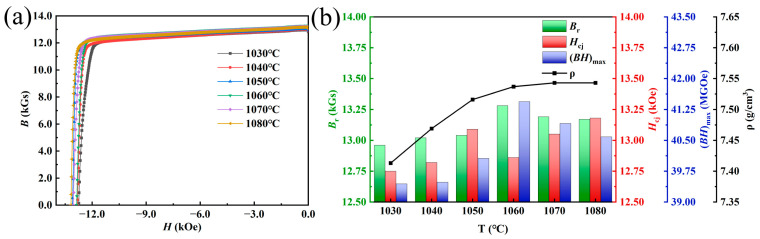
(**a**) Room-temperature demagnetization curves of magnets with different sintering temperatures; (**b**) plots of remanence, coercivity, maximum magnetic energy product, and density versus sintering temperature.

Figure 1 shows the demagnetization curves of magnets with different sintering temperatures at room temperature, as well as the trends of magnetic properties and densities. The specific values of magnetic properties and densities of the corresponding magnets are shown in Table 1. It can be seen from the figure that with the increase in sintering temperature, the density and magnetic properties of the magnet increase simultaneously. When the sintering temperature reached 1060 °C, the magnet density reached 7.537 g/cm^3^, and the remanence and the maximum magnetic energy product also reached the maximum (13.28 kGs and 41.44 MGOe), but the coercivity showed an abnormal decrease. With the further increase in sintering temperature, the change in magnet densities tended to be gentle, the remanence and the maximum magnetic energy product decreased, and the coercivity increased again and reached its maximum value (13.18 kOe) at 1080 °C.

**Figure 2:**
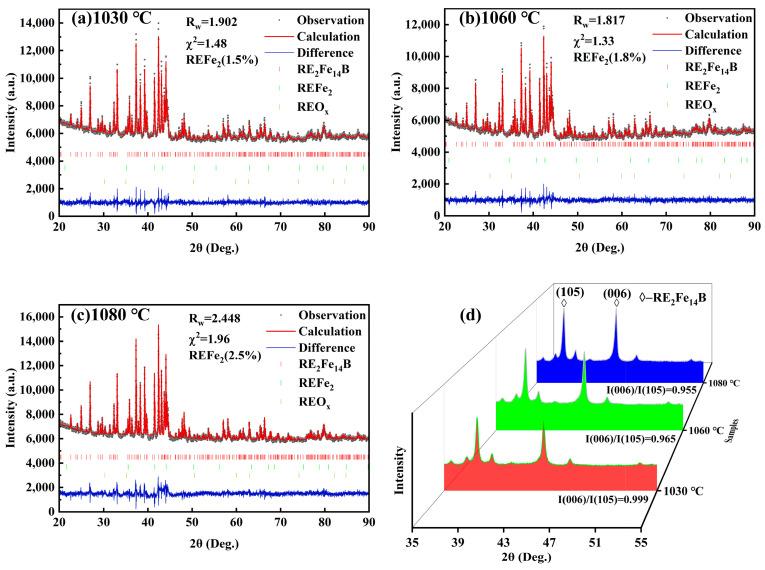
Powder XRD spectra (**a**–**c**) and bulk XRD spectra (**d**) of sintered magnets at 1030 °C, 1060 °C and 1080 °C.

In order to understand the intrinsic correlation between the magnetic properties and the sintering temperature, XRD was used to determine the information about the phase structure of the magnet under different sintering temperatures (Figure 2), and the corresponding results of the structural refinement are shown in Table 2. The results show that the magnet is dominated by the RE_2_Fe_14_B phase with a small amount of REFe_2_ phase. The proportion of REFe_2_ in the magnet increases from 1.5% to 2.5% with increasing sintering temperature. The structural parameters of RE_2_Fe_14_B are also affected by the sintering temperature. As the temperature increases from 1030 °C to 1080 °C, the cell parameter a/c increases from 8.78789/12.19693 Å to 8.78874/12.20015 Å, which involves the compositional and structural compatibility of the element with the main phase and the grain boundary phase, and the element Ce is more inclined to enter the grain boundary to form CeFe_2_ phase at high temperatures.

The sintering temperature also causes a significant difference in the magnet orientation. As the sintering temperature increases from 1030 °C to 1080 °C, the intensity ratio of (006)/(105) diffraction peak decreases from 0.999 to 0.955, and the magnetic orientation decreases.

According to the magnet’s remanence Equation (1):

(1) $$\begin{array}{c} B_{r}=A\bar{cos\theta }\left(1-\beta \right)\frac{\rho }{\rho_{0}}M_{s}\# \end{array}$$

The remanence is multiplied by the ratio of the main phases, the degree of orientation, and the densification. When the sintering temperature is low (1030 °C), the densification of the magnet plays a dominant role in the remanence, the high main phase ratio and orientation have less influence on the remanence, and the remanence is poor. When the sintering temperature is increased to 1060 °C, the remanence reaches the maximum value, at which time the densification tends to stabilize, and the influence on the remanence gradually becomes smaller. Further increases in the sintering temperature lead to an increase in the proportion of REFe_2_ phase in the magnet and a decrease in the viscosity of the RE-rich liquid phase, which in turn leads to the rotation of the main phase grains, deterioration in grain orientation, and a significant decrease in remanence [24].

**Figure 3:**
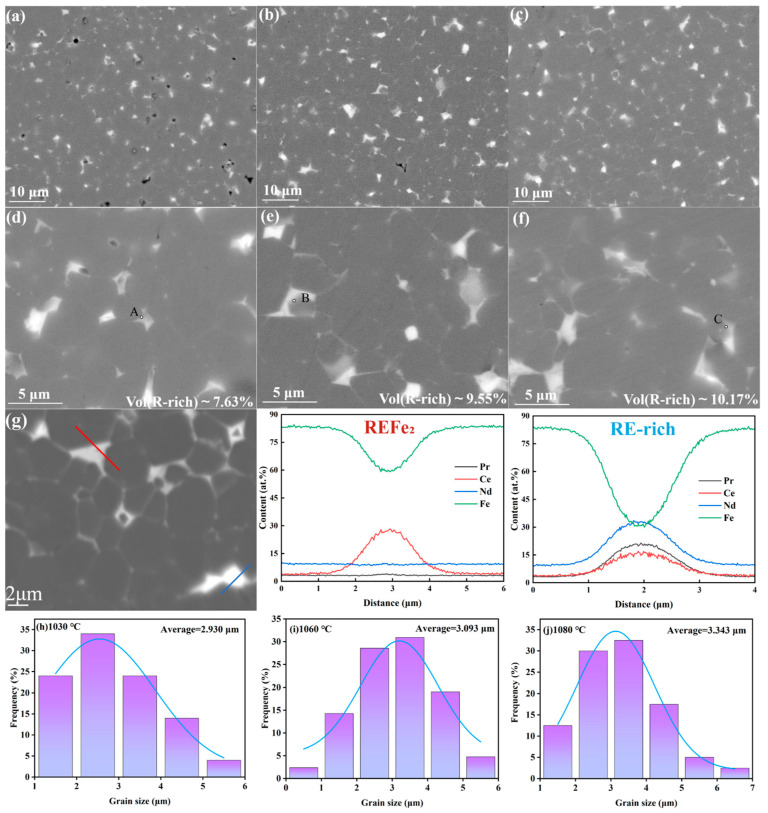
Backscattered electron scanning electron microscopy (SEM) images of sintered magnets at 1030 °C (**a**,**d**), 1060 °C (**b**,**e**), and 1080 °C (**c**,**f**). (**g**) Scanning magnification of the magnets at 1080 °C and line scans of the REFe_2_ phase and the RE-rich phase, as well as grain sizes of the sintered magnets at 1030 °C (**h**), 1060 °C (**i**), and 1080 °C (**j**).

The microstructure morphology of the magnets at different sintering temperatures was observed using backscattered electron scanning electron microscopy, as shown in Figure 3. Figure 3a,c show SEM images of magnets under 5000× magnification at different sintering temperatures. There are many holes and other defects in the magnet with a sintering temperature of 1030 °C, and most of the main phase grains are in direct contact with each other. However, the magnets with sintering temperatures of 1060 °C and 1080 °C have no hole defects, and there are very thin grain boundary layers between the main grains. Figure 3d,f show SEM images of magnets under 15,000× magnification at different sintering temperature. At higher magnification, the grain boundary phase ratio of the magnet sintered at 1030 °C is lower (7.63%), and RE-rich phase agglomeration is obvious, and the continuity is poor. As the sintering temperature increased to 1060 °C, the proportion of grain boundary phase increased (9.55%), and the phenomenon of grain boundary agglomeration decreased and was distributed uniformly and continuously around the main phase. The further increase in the sintering temperature (1080 °C) increased the proportion of the grain boundary phase to 10.17%, the uniformity and continuity of the distribution of the grain boundary phase were further optimized, and the main phase grains became rounded and smooth.

The low sintering temperature affects the liquid-phase sintering process, resulting in the poor flow of the rare-earth phase, poor liquid-phase wetting of the main-phase particles, poor shrinkage of the magnet, and relatively low densities (7.413 g/cm^3^), which seriously affects the magnetic properties of the magnets (*B*_r_ = 12.96 kGs, *H*_cj_ = 12.75 kOe). With the increase in sintering temperature, the grain boundary phase mobility of the magnet increases, and the better wettability makes the holes inside the magnet fill with liquid phase, the density increases rapidly (7.537 g/cm^3^), the continuity distribution of the grain boundary improves, the exchange coupling between the main phase grains is effectively reduced, and the remanence and coercivity are improved. However, the increase in sintering temperature will also lead to the increase in magnet grain size, as shown in Figure 3h–j. According to the expression of coercive force with grain size summarized by Li et al. [25], *H*_cj_ = a-bln(D^2^) is not conducive to the coercive force of magnets. When the sintering temperature is less than 1060 °C, the hole defect is the main factor affecting the coercivity, which gradually increases with the increase in sintering temperature. When the sintering temperature reaches 1060 °C, the effect of pores on coercivity decreases, and the growth of grain becomes the main factor limiting coercivity. However, when the sintering temperature exceeds 1060 °C, the coercivity increases instead of decreasing, which is related to the optimization of the internal structure of the magnet. In other words, the high-temperature sintering causes the main phase to dissolve, which makes the original angular main phase grains become round and smooth, the main phase ratio increase, and the reverse magnetizing domain nucleation be difficult.

As shown in Table 3, EDS point scanning was used to test the edge of ther RE_2_Fe_14_B main phase components at different sintering temperatures to analyze the reasons for the optimization of magnet performance. With the increase in sintering temperature, the proportion of Ce element at the edge of the main phase decreases, indicating that Ce diffuses from the main phase to the grain boundary phase. The microstructure and morphology of the magnet sintered at 1080 °C were further analyzed, as shown in Figure 3g. The white part is the RE-rich phase (blue line), and the gray part is the REFe_2_ phase (red line), which is in good agreement with the XRD results. The former is a conventional intergranular phase, while the latter exists in cerium containing magnets as a grain boundary phase, which affects the properties of the magnets.

**Figure 4:**
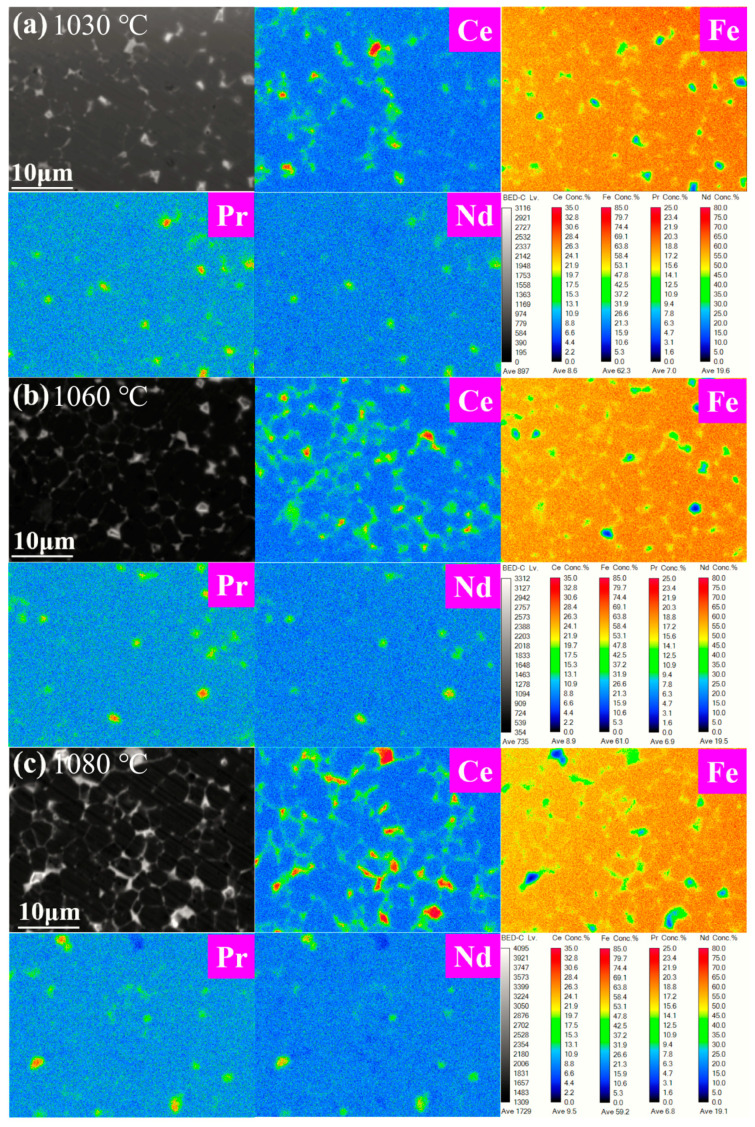
EPMA maps of 1030 °C (**a**), 1060 °C (**b**), and 1080 °C (**c**) magnets.

Figure 4 shows the EPMA mapping of the magnets at different sintering temperatures. The results show that with the increase in sintering temperature, the proportion of the grain boundary phase increases, and the grain boundary becomes continuously smooth and wrapped around the main phase, which is consistent with the SEM results. The distribution of the Ce element reflects strong sensitivity to sintering temperature. When the temperature is low (1030 °C), the agglomeration of the Ce element is obvious, and a large amount of Ce element is found in the main phase grain epitaxial layer. With the increase in sintering temperature, the distribution uniformity of Ce is optimized, and the concentration of Ce in the main phase decreases. When the temperature reaches 1080 °C, the Ce element is enriched at the grain boundary and shows good continuity, and the Ce and Fe elements overlap with each other. The results show that the precipitation of CeFe_2_ phase at the grain boundary increases with the increase in sintering temperature, which is consistent with the XRD results, which is also an important reason for the improvement of the magnetic grain boundary phase.

**Figure 5:**
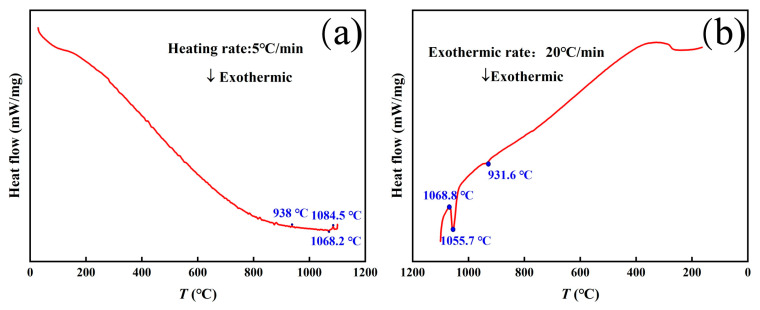
DSC curves of magnet warming up (**a**) and cooling down (**b**).

Figure 5 shows the DSC curves of the magnets at different rates of temperature rise and fall. At a heating rate of 5 °C/min, the magnet undergoes a phase transition reaction of CeFe_2_ at 938 °C, which proceeds as CeFe_2_ + Ce_2_Fe_14_B ↔ Ce_2_Fe_17_ + L. The CeFe_2_ phase melts in the high-temperature sintering process, and a liquid phase is added to the conventional RE-rich phase to optimize the density of the magnet and the wettability of the main phase, which weakens the magnetic exchange coupling effect of the main phase and improves the coercivity of the magnet. When the temperature ranges from 1068.2 °C to 1084.5 °C, the magnetic phase transition reaction occurs again, and the reaction process is Ce_2_Fe_14_B + Ce_2_Fe_17_ ↔ L + γ-Fe. This also fully confirms that when the sintering temperature of the magnet exceeds 1068.2 °C, the pre-sintered Ce_2_Fe_17_ phase further decomposes to form more liquid phase, thereby improving the grain boundary phase ratio and continuity, which is also the reason for the increase in the coercivity of the magnet. The 20 °C/min cooling DSC curves at 931.6 °C, 1055.7 °C, and 1068.8 °C in Figure 5b well confirm the existence of the two phase transition intervals, but the non-equilibrium solidification occurs due to the fast cooling rate, resulting in a slight decrease in the reaction temperature.

## 4. Conclusions

In this study, the relationship among the sintering temperature, phase structure, and magnetic properties of Ce-containing NdFeB magnets was investigated.

1. The sintering temperature affects the liquid phase ratio and element mobility and improves the microstructure and magnetic properties of the magnet. At a 1080 °C sintering temperature, the magnet *B*_r_ = 13.17 kGs, *H*_cj_ = 13.18 kOe, and (*BH*)_max_ = 40.58 MGOe.

2. The remanence is closely related to the sintering temperature. When the sintering temperature is low (1030 °C), the densification of magnet plays a leading role in the remanence. With the increase in temperature, the densification of the magnet tends to be stable, and the ratio of the principal phase and orientation have a decisive effect on the remanence.

The coercivity of Ce-containing magnets increases at a high temperature (1080 °C). When the sintering temperature is low (1030 °C), the fluidity of the grain boundary phase is poor, resulting in the strong exchange coupling formed by the direct contact of the main phase, and the coercivity is low (*H*_cj_ = 12.75 kOe). With the increase in temperature, the influence of grain growth on the magnet decreases, and the increase of the CeFe_2_ phase ratio (2.5%) causes the magnet to form more liquid phase. This improves the continuity and wettability of the grain boundary, weakens the magnetic exchange coupling of the main phase, and increases coercivity.

## Figures and Tables

**Table 1:** Room-temperature magnetic properties of magnets with different sintering temperatures.

T (°C)	*B*_r_ (kGs)	*H*_cj_ (kOe)	(*BH*)_max_ (MGOe)	*H*_k_/*H*_cj_ (%)	ρ (g/cm^3^)
1030	12.96	12.75	39.44	93.7	7.413
1040	13.02	12.82	39.48	95.7	7.469
1050	13.04	13.09	40.06	96.8	7.516
1060	13.28	12.86	41.44	95.8	7.537
1070	13.19	13.05	40.91	95.8	7.543
1080	13.17	13.18	40.58	95.8	7.543

**Table 2:** Lattice parameters, R_w_ and χ^2^, of magnets analyzed by Rietveld at 1030 °C, 1060 °C, and 1080 °C.

T (°C)	a (Å)	c (Å)	REFe_2_ (%)	REO_x_ (%)	R_w_	χ^2^
1030	8.78789(2)	12.19693(2)	1.5	0.9	1.902	1.48
1060	8.78825(2)	12.19958(2)	1.8	1.2	1.817	1.33
1080	8.78874(2)	12.20015(2)	2.5	0.7	2.448	1.96

**Table 3:** EDS results at 1030 °C (A), 1060 °C (B), and 1080 °C (C).

Atomic Percent (%)	Fe	Ce	Pr	Nd
A	82.2	8.45	2.22	7.13
B	82.75	8.13	2.17	6.95
C	82.51	6.83	2.69	7.96

## Data Availability

The original contributions presented in the study are included in the article, further inquiries can be directed to the corresponding authors.
